# News and Events of the Week

**Published:** 1909-12-25

**Authors:** 


### News and Events of the Week.

The estate of the late Dr. Herbert Groves Lee, M.D., of The Moats, Thame, Oxon, has been valued at £23,392 gross and £19,271 net.

The estate of Dr. John Milson Rhodes, of Didsbury, Manchester, who died on September 25, aged 62, has been valued at £657 gross and £520 net.

Colonel George McBride Davis, M.D., C.B., D.S.O., lately principal medical officer of the Punjab Frontier Force and of the Expeditionary Forces in China, who died on October 4, aged sixty-three, left estate valued at £16,799 gross and £16,677 net.

According to the *Poor Law Officers' Journal*, inquiry was made of the Local Government Board as to whether fees for vaccination should be regarded as emoluments of the office of a workhouse medical officer within section 10 of the Poor Law Officers Superannuation Act, 1896 (59 and 60 Vict. c. 50). In reply, the Board stated that where in pursuance of the Vaccination Order, 1898, a contract for vaccination is entered into with the medical officer of the workhouse, or such a contract entered into before the commencement of the Order is continued in force, they think that the effect of the Vaccination Order is to make the fees under the contract with the medical officer an emolument within the meaning of the section.

The Royal College of Surgeons of Edinburgh were fortunate in securing Lord Rosebery as their principal guest at the annual dinner of the College on December 14. Before opening the new hall and museum Lord Rosebery said that a forebear of his—Gilbert Prymrose—was the first recorded Fellow of the College of Surgeons in Edinburgh. In his hand was the mortar which Gilbert Prymrose employed when he pounded the gruesome drugs which he administered to the unfortunate population. He was justly proud of his connection with the old College of Surgeons by Gilbert Prymrose and by his son Duncan Prymrose, who was a surgeon of the Kings of Scotland. It was in 1505 that the College received its first charter, which was discreetly limited to those who could both read and write. (Laughter.) What was interesting to him and to all interested in the history of their country was that all these great institutions with which the fame of Scotland was indissolubly linked—the Universities, the College of Surgeons, and other learned bodies of that kind—all came into existence in a time of storm and stress. Their travail was the rudest in the world. They were produced at a time when Scotland could hardly keep her national existence. Might he say one word of the gratitude that they owed to the practice of surgery itself? There was a beautiful saying about surgeons which he supposed was familiar, but which some laymen did not know. The great surgeon required the hand of a lady and the heart of a lion. (Cheers.) There was one surgeon to whom the whole universe owed a debt of gratitude, and he was a Fellow of the College of Surgeons of Edinburgh. He asked them to allow him to propose the toast and to open the hall, two tasks which he had to combine, by giving them the name of Lord Lister. (Loud cheers.) The President, Mr. J. Montagu Cotterill, replied, and intimated that at a meeting of the College on December 10 it was unanimously agreed that Lord Rosebery be elected an honorary Fellow of the College.

The medical officer of health for the various Councils comprised in the Mid-Warwickshire Joint Sanitary District has officiated in that capacity for the Crick Rural District Council. Consequent, however, on the withdrawal of Yardley from the combination and the resignation of Dr. Wilson, the Crick Council has to make another appointment, and it is the wish of several members that a doctor resident in the district should be appointed. The Local Government Board was written to on the matter, and at a meeting of the Council its reply was read to the effect that the employment of a general practitioner as medical officer of health was not desirable where a medical man possessing a diploma in public health or having previous training in public health work and who is debarred from private practice could be appointed. It was stated there was a strong feeling in the district in favour of the appointment of a local doctor, and the clerk was directed to ask if the Local Government Board would continue to pay half the salary if a local man were appointed.

Three new lectureships were instituted by the Council of the University at their meeting on November 30 on the recommendation of the Senate and of the Faculty of Medicine: (1) A lectureship in orthopædic surgery, to which Mr. Robert Jones, Ch.M.Liv., F.R.C.S.Edin., was appointed. (2) A lectureship in physiology, to which the present assistant lecturer and demonstrator, Dr. H. E. Roof, was appointed. (3) A lectureship in pharmacology, to which Owen T. Williams, M.B.Lond., M.R.C.P., was appointed. The lecturer in orthopædics will deliver a course of lecture-demonstrations during the summer term, which will be open to graduates and undergraduates alike. The lecturer in physiology will take special charge of the Chemical Physiology Department. The lecturer in pharmacology was temporary lecturer in the subject during the last two sessions; he will supervise pharmacological research and will hold a class in practical pharmacology, which is in the third year of the curriculum for the degree.

### THE CHILD-STUDY SOCIETY.

The Council of the Child-Study Society has for some time, in their efforts to advance our knowledge of child life, been looking for aid of a scientific character, and they approached Professor Karl Pearson, F.R.S., who with his equipment and laboratory at University College, London, is in a position to render exceptional service. Professor Pearson has drafted a schedule for studying the factors influencing the social life of the child, which he desires to have filled in by heads of families, or by teachers intimate with families. The number in the family need not be large, but particulars of father, mother, and at least two children are required. It is more important that the schedule should be filled up for families "of the upper middle or professional classes." In view of the widespread interest now taken by parents in the more scientific treatment of child-life, we trust that a good response will be forthcoming. Parents who would prefer that the actual names of their family should be withheld, can fill up a copy of the schedule on that understanding, supplying their name solely to the secretary who issues the copy with a key number. Copies can be obtained from the Secretary of the Child-Study Society, London, 90 Buckingham Palace Road, London, S.W.

